# Non-Enzymatic Glucose Sensors Composed of Polyaniline Nanofibers with High Electrochemical Performance

**DOI:** 10.3390/molecules29112439

**Published:** 2024-05-22

**Authors:** Nebras Sobahi, Md. Mottahir Alam, Mohd Imran, Mohammad Ehtisham Khan, Akbar Mohammad, Taeho Yoon, Ibrahim M. Mehedi, Mohammad A. Hussain, Mohammed J. Abdulaal, Ahmad A. Jiman

**Affiliations:** 1 Department of Electrical & Computer Engineering, Faculty of Engineering, King Abdulaziz University, Jeddah 21589, Saudi Arabia; nsobahi@kau.edu.sa (N.S.);; 2 Department of Chemical Engineering, College of Engineering, Jazan University, Jazan 45142, Saudi Arabia; 3 Department of Chemical Engineering Technology, College of Applied Industrial Technology, Jazan University, Jazan 45142, Saudi Arabia; 4 School of Chemical Engineering, Yeungnam University, Gyeongsan-si 38541, Gyeongbuk-do, Republic of Korea; 5 Center of Excellence in Intelligent Engineering Systems (CEIES), King Abdulaziz University, Jeddah 21589, Saudi Arabia

**Keywords:** electrochemical detection, glucose, polyaniline, fiber, sensing

## Abstract

The measurement of glucose concentration is a fundamental daily care for diabetes patients, and therefore, its detection with accuracy is of prime importance in the field of health care. In this study, the fabrication of an electrochemical sensor for glucose sensing was successfully designed. The electrode material was fabricated using polyaniline and systematically characterized using scanning electron microscopy, high-resolution transmission electron microscopy, X-ray diffraction, Fourier transform infrared spectroscopy, and UV-visible spectroscopy. The polyaniline nanofiber-modified electrode showed excellent detection ability for glucose with a linear range of 10 μM to 1 mM and a detection limit of 10.6 μM. The stability of the same electrode was tested for 7 days. The electrode shows high sensitivity for glucose detection in the presence of interferences. The polyaniline-modified electrode does not affect the presence of interferences and has a low detection limit. It is also cost-effective and does not require complex sample preparation steps. This makes it a potential tool for glucose detection in pharmacy and medical diagnostics.

## 1. Introduction

According to a recent survey by the WHO, more than 400 million people around the world suffer from diabetes [1]. The main symptom of diabetes is hyperglycemia, which may lead to chronic damage and dysfunction of body organs [2,3]. Diabetes is determined by the glucose concentration in blood and urine; therefore, its detection with a high sensitivity and user-friendly approach is of prime importance [4]. There is also a great demand for glucose monitors in the food and chemical industry. Researchers have developed many methods of detection, including flow cyclometric, liquid chromatography, mass spectroscopy, and fluorescence [5,6,7,8]. However, these methods have limitations, such as low accuracy, high cost, and complexity of operation. Therefore, the design of a highly sensitive, cost-effective, and simple technique is the need of the hour. Recently, electrochemical-based sensors have received a lot of attention from researchers due to their rapid, simple, and cheap way [9,10,11,12,13]. Electrochemical sensing has emerged as a powerful and versatile technique with widespread applications in various fields, ranging from healthcare and environmental monitoring to industrial process control and food safety assessment [14,15,16,17,18,19,20]. The significance of electrochemical sensing stems from its unique capabilities to detect and quantify analytes with high sensitivity, selectivity, and reliability, coupled with the advantages of rapid response, ease of miniaturization, and cost-effectiveness [21,22].

Nanoparticles are commonly employed in the manufacture of electrodes for chemical sensors and biosensors and play a significant role in electrochemical detection [23,24,25,26,27]. Nanostructured materials are widely used in a wide range of applications, including heat transfer, photocatalysis, hydrogen production, solar cells, biomedical equipment, and therapies, in addition to electrochemical sensing applications [27,28,29,30,31,32]. Numerous electroactive materials have contributed to this technology for decades and worked effectively in electrochemically based sensors. The electrochemical activity of various nanomaterials in terms of sensitivity, selectivity, accuracy, and response time has been investigated [33,34]. Current glucose-sensing methodologies predominantly rely on enzymatic approaches, which, despite their widespread use, are plagued by limitations such as susceptibility to interference, operational instability, and high production costs. Traditional glucose sensing methodologies primarily rely on enzymatic reactions catalyzed by glucose oxidase or glucose dehydrogenase enzymes immobilized onto electrodes [35,36]. While enzymatic sensors offer high specificity and sensitivity, they are often limited by issues such as enzyme instability, susceptibility to interference, and high production costs [37,38,39]. In response to these pressing challenges, our research focuses on the development of a novel non-enzymatic glucose sensor platform based on polyaniline nanofibers.

The various nanostructures of polyanilines have been reported as electrochemical electrode materials, including nanoparticles, nanosheets, and nanotubes [40,41]. However, the nanofiber nanostructure has the advantage of being a porous structure that provides more active sites for redox reactions [42]. In other studies, researchers have also explored different nanostructured materials for various applications [42,43,44,45,46,47,48,49,50,51,52,53]. Materials in the nanoscale often display distinct physical, chemical, and biological characteristics that deviate from those of their larger counterparts. These qualities that vary depending on size might be used for specific applications [54,55,56,57,58].

A sensitive and specific electrochemical electrode modified with polyaniline nanofibers was developed to sense the glucose. The obtained polyaniline-modified sensor showed enhanced electrocatalytic activity toward glucose. The fabricated electrode was tested on various parameters, including sensitivity and stability. This innovative approach capitalizes on the unique physicochemical properties of PANI-NF to achieve superior sensing performance, including high sensitivity, selectivity, and long-term stability. By leveraging nanotechnology and advanced electrochemical techniques, our sensor platform holds the promise of revolutionizing glucose monitoring paradigms and improving patient outcomes in diabetes management.

## 2. Results and Discussion

**Figure 1:**
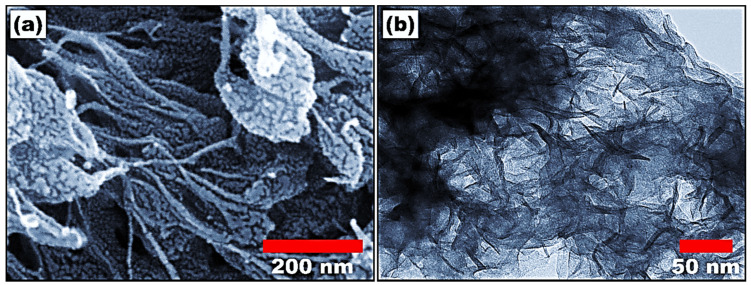
(**a**) Field emission scanning electron microscope and (**b**) high-resolution transmission electron microscope image of polyaniline nanofibers.

The morphology of synthesized polyaniline was investigated using field emission scanning electron microscopy (FESEM), as shown in Figure 1a. The FESEM image shows the nanofiber morphology of polyaniline. The fibers are randomly oriented, giving the illustration of a three-dimensional structure. The average length and diameter of the nanofiber are calculated to be 500 nm and 20 nm, respectively. The formation of nanofibers shows the homogeneous nucleation of the polyaniline. The morphology of polyaniline was further studied using high-resolution transmission electron microscopy (HR-TEM), as shown in Figure 1b. The HR-TEM images match the FESEM results and show the fiber-like morphology of polyaniline. The fiber-like morphology could increase the surface area of the material, which improves the detection ability.

**Figure 2:**
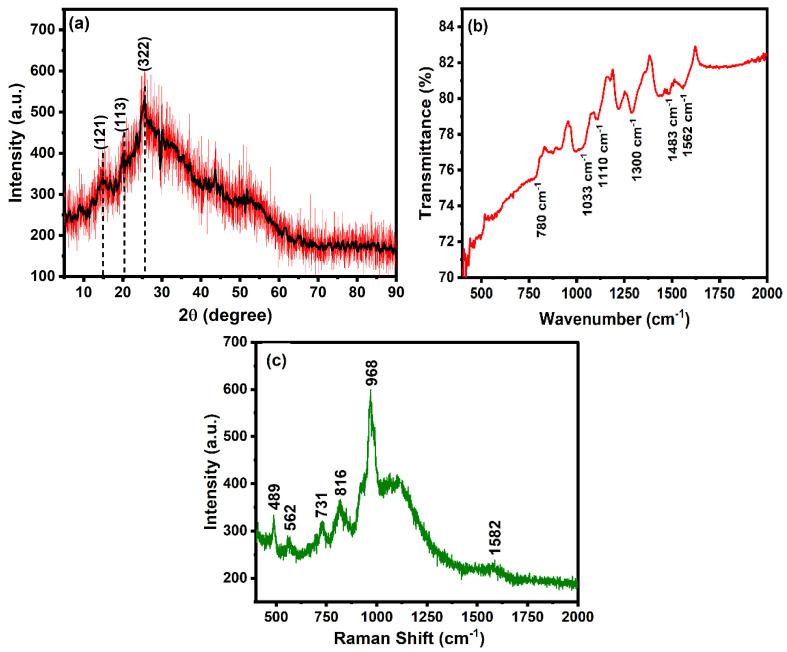
(**a**) XRD diffractogram, (**b**) FTIR spectrum, and (**c**) Raman spectrum of polyaniline.

The structural aspect of the polyaniline was studied using an XRD diffractogram, as shown in Figure 2a. The XRD diffractogram shows the dominant peak at 25.6°, corresponding to the (322) plane, which is the characteristic peak of polyaniline [59]. The diffraction peaks located at 2*θ*, 4.8° and 20.3°, correspond to the (121) and (113) planes, respectively. The sharp peaks observed in XRD patterns show the semi-crystalline nature of polyaniline. The interplanar spacing was calculated using the following equations.

(1) Bragg’s Law: nλ=2dsinθ

where *n*, *λ*, *d*, and *θ* correspond to the order of diffraction, the wavelength of the X-rays, interplanar spacing, and diffraction angle, respectively. The interplanar spacing in the polyaniline is calculated to be 0.33 nm using a value of *n* = 1 and the wavelength of X-rays as 1.54 Å.

The crystallite size was calculated using the Scherer equation

(2) $$D=\frac{k\lambda }{\beta cos\theta }$$

where *D* = average crystallite size, *β* = line broadening in radians, *θ* = Bragg angle, *λ* = X-ray wavelength. The calculated average crystallite size is found to be 0.36 nm.

FTIR spectrum of polyaniline in the range of 400–2000 cm^−1^ is shown in Figure 2b. The C–H stretching vibration of a benzene ring and C-Cl group can be assigned to the band located at 780 and 1033 cm^−1^, respectively [60]. The FTIR bands situated at 1300 and 1110 cm^−1^ are attributed to the C–N benzenoid and C–H stretching vibration of polyaniline, respectively [60]. The FTIR peaks located at 1562 and 1483 cm^−1^ correspond to C=N and C=C stretching vibration modes of benzenoid of polyaniline, respectively [61,62]. The characteristic bonds on the surface of the nanomaterial confirm the formation of polyaniline.

The electrochemical detection of glucose takes place on the surface of polyaniline. The oxidation is started by the interaction of glucose on polyaniline. The glucose interacts with polyaniline via weak Van der Waals interactions, hydrogen bonding, and electrostatic interactions. The electronegative functional groups present on the surface of polyaniline (C–N) form hydrogen bonds with hydrogen groups of glucose [63].

The Raman spectrum using a 785 nm excitation source was recorded to study the vibrational mode of polyaniline. As observed in Figure 2c, the bands appeared in the range of 400–2000 cm^−1^, corresponding to different stretching and vibrational modes. The bands in the range of 400–1100 cm^−1^ correspond to the deformation vibration of benzene rings [64]. The band located at 816 cm^−1^ is attributed to the in-plane vibration of protonated polyaniline. The band located at 1582 cm^−1^ corresponds to the stretching vibration of the quinonoid. The presence of characteristic bands of polyaniline confirms the successful synthesis.

**Figure 3:**
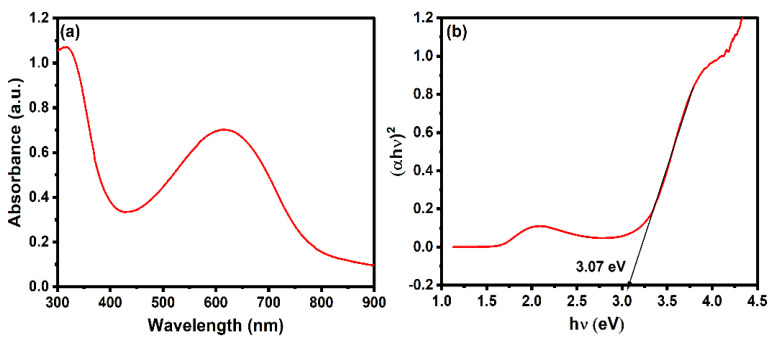
(**a**) Absorbance spectrum and (**b**) Tauc’s plot of polyaniline.

The optical properties of the polyaniline have been studied using UV-visible spectroscopy. As shown in Figure 3a, the polyaniline has an absorbance in the range of 300–900 nm. The characteristic peak at 300 and 600 nm confirms the formation of polyaniline. The broad peaks located at ~320 nm and ~600 nm are attributed to the polaron and bipolar on-band transition, respectively [65]. As shown in Figure 3b, the band gap of the polyaniline was determined by Tauc’s plot using the following equation:

(3) $${\left(\alpha h\nu \right)}^{n}=A\left(h\nu -E_{g}\right)$$

where *α*, *h*, *v*, and *E_g_* correspond to the absorption coefficient, Planck’s constant, frequency of the light, and band gap of the material. The band gap was calculated by plotting ${\left(\alpha h\nu \right)}^{n}$ vs. hν and extrapolation of the linear region will intercept the x-axis to give the value of the band gap. The value of *n* is ½ and 2 for indirect and direct bandgap transition. The band gap of the polyaniline, considering the direct band gap transition, was found to be 3.07 eV [66]. The band gap of the polyaniline lies in the visible range.

**Figure 4:**
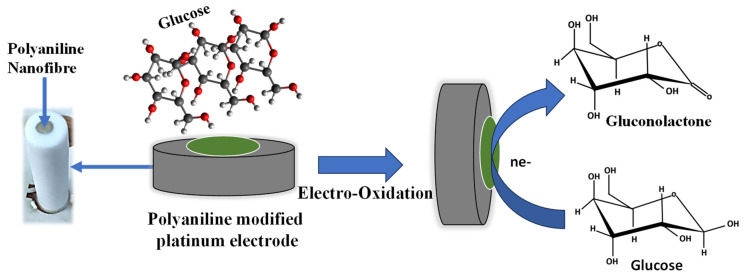
The schematic for the electrochemical mechanism for sensing glucose.

The electrochemical sensing mechanism of glucose using polyaniline nanofiber is shown in Figure 4. Initially, the glucose molecules from the solution adsorb onto the surface of the electrode. The absorbed glucose undergoes electrochemical oxidation in the presence of an applied potential. At the anodic (positive) electrode, glucose molecules lose electrons, resulting in the formation of gluconate ions and the release of protons. The electrochemical oxidation of glucose results in the generation of an electrical current, which can be measured using an external circuit. The chemical reaction for the glucose oxidation in the electrode is as follows:

(4) $$C_{6}H_{12}O_{6}\to C_{6}H_{11}O_{7}^{-}+2H^{+}+2e^{-}$$

**Figure 5:**
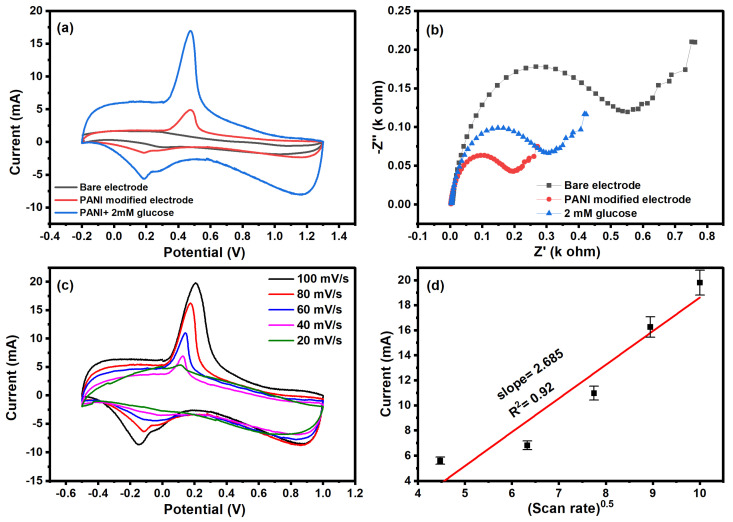
(**a**) Cyclic voltammetry, (**b**) Nyquist plot of bare, modified electrode and in the presence of glucose, (**c**) cyclic voltammetry at different scan rates, and (**d**) plot between the current and square root of scan rate.

The CV scan of the bare electrode, polyaniline-modified electrode, and modified electrode in the presence of glucose is shown in Figure 5a. The bare electrode shows no dominant peak, confirming there is no redox reaction on the surface of the electrode. However, the polyaniline-modified electrode shows an oxidation peak in the given potential range. The CV scan of the modified electrode in the presence of glucose (2 mM) shows an increase in the oxidation peak, confirming the electrochemical detection of glucose. In the electrochemical detection of glucose, the oxidation of glucose leads to an increase in the current. The oxidation peak in CV is attributed to the oxidation of glucose into gluconolactone.

We observed the change in electron transfer resistance of the bare electrode, modified electrode, and electrode in the presence of glucose, as shown in Figure 5b. The oxidation of glucose changes the charge distribution as well as the mobility of ions at the diffusion layer, resulting in reduced resistance. All three plots show semi-circle and linear behavior at lower and higher frequency ranges that are attributed to diffusion-limited electron transfer. The semi-circular region indicates the charge transfer mechanism between the electrode and electrolyte. The modified electrode has a smaller resistance compared to the bare electrode, confirming the low charge transfer on the surface of polyaniline. The polyaniline has low resistance due to the polaron delocalization along the chain. In the presence of glucose, the resistance of the electrode was further decreased; this was attributed to absorbed glucose, which enhanced the electron transport on the polyaniline surface. The influence of scan rate on the electrochemical behavior was investigated, as shown in Figure 5c. The CV plot at different scan rates shows the decrease in reduction peak current with an increase in scan rate. The electrochemical kinetics of the detection were studied by plotting between the square root of the scan rate and reduction peak current, as shown in Figure 5d. The linear relationship of the graph confirms the diffusion-controlled electrochemical process in the detection of glucose with a slope of 2.685 × 10^−3^ and a correlation coefficient of 0.92.

**Figure 6:**
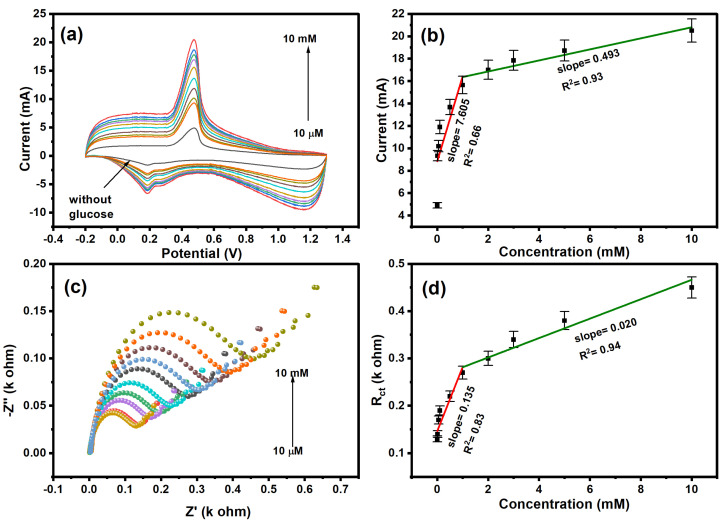
(**a**) CV and (**c**) EIS plot of glucose at different concentrations. Calibration curve with the linear equation plotted for (**b**) CV and (**d**) EIS.

The electrochemical performance was studied for different concentrations (10 µM–10 mM) of glucose, as shown in Figure 6a. The cyclic voltammetry shows a decrease in redox peak current with a decrease in glucose concentration. The high current was observed at higher concentrations due to the availability of redox probe ions toward the surface of polyaniline [67]. As shown in Figure 6b, the calibration curve between the log of the concentration of glucose and redox current was plotted, and the calibration curve exhibits two linear ranges with a correlation coefficient of 0.66 and 0.93. At low concentrations of glucose (0–0.5 mM), the rate of diffusion in polyaniline is faster with rate kinetics (k_1_ = 7.605), and the linear range shows that the electrode is not saturated yet. However, at higher concentrations (1–10 mM) of glucose, the activated surface area of the polyaniline-modified platinum electrode decreased due to the accumulation of glucose molecules that tended to saturation of the sensor with rate kinetics (k_2_ = 0.493). The Nyquist plot of the polyaniline-modified electrode in the concentration range of (10 µM–10 mM) glucose is shown in Figure 6c. The resistance of the electrode increased with increasing concentrations of glucose. This is attributed to the poor charge transfer of ions at the electrode–electrolyte interface [67]. The polyaniline-modified electrode shows a higher charge transfer of 0.45 kΩ at a 2 mM concentration of glucose. As shown in Figure 6d, the calibration plot between the log of concentration of glucose and resistance shows a linear relationship with a correlation coefficient of 0.83 and 0.94. The limit of detection was calculated using the formula: (3 × SD)/s, where SD is the standard deviation, and s is the slope of the calibration curve. The calculated limit of detection is found to be 10.6 µM.

As observed from Table 1, this work provides an improved detection range and lower detection limit for the non-enzymatic electrochemical detection of glucose. The polyaniline nanostructured material offers a high surface area-to-volume ratio, facilitating greater analyte interaction and leading to enhanced sensitivity. Additionally, the unique morphology of PANI nanofibers promotes efficient electron transfer, contributing to improved sensor response kinetics.

**Figure 7:**
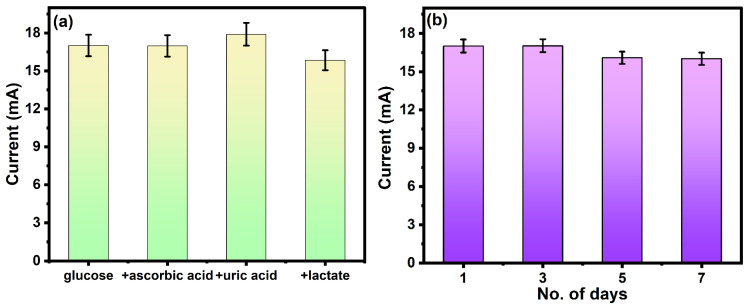
(**a**) Selectivity of the electrode in the presence of interference and (**b**) the stability of the same electrode for 7 days.

The sensitivity and stability are important parameters for the investigation of the electrochemical performance of the sensor. The stability or repeatability of the electrode is measured by the absence of incompatibility between consecutive measurements by the same electrode. The effect of interferences was analyzed on the fabricated electrode by performing the electrochemical detection of glucose in the presence of interference such as uric acid, lactate, and ascorbic acid. The concentration of glucose was fixed (2 mM), and the concentration of interferences was 10 mM. As shown in Figure 7a, the oxidation peak remains unaffected by the interferences. This is an important parameter for real-life applications because the signal will be less sensitive to interfering species and will be effective for the detection of glucose.

The stability of the electrode was tested by scanning the same electrode for 7 days, as shown in Figure 7b. The redox current shows negligible change up to 7 days, which confirms the promising stability of the electrode. Repetitive measurements indicated that this electrode has good reproducibility and does not undergo surface fouling during the voltammetric measurements. PANI nanofibers exhibit superior resistance to degradation, making them well-suited for long-term sensing applications. This inherent stability ensures consistent sensor performance over extended periods.

## 3. Materials and Methods

### 3.1. Materials

Aniline, with a purity of >99.5%, was bought from Sigma Aldrich in St. Louis, Mo, USA. Hydrochloric acid (HCl) with >37% purity was purchased from Chem-Lab in Zedelgem, Belgium. A >98% pure ammonium persulfate was bought from Fisher Scientific, Chennai, TN, India. The Puris-Expe water system was utilized to obtain the deionized (DI) water used in this project. The compounds were utilized in their original form.

### 3.2. Synthesis of Polyaniline

**Figure 8:**
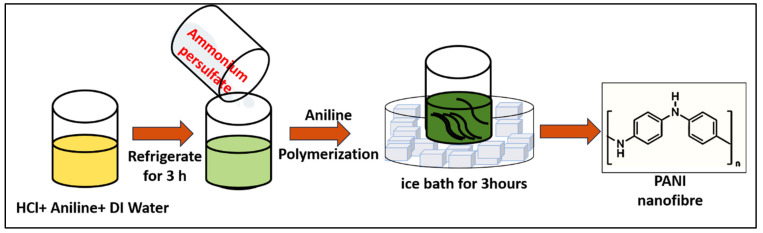
The schematic for the synthesis of polyaniline by polymerization method.

In this study, the polyaniline was synthesized using the chemical oxidative polymerization method with aniline and ammonium persulfate as monomer and initiator, respectively. Oxidative polymerization is an easy and inexpensive method for the synthesis of polyaniline in bulk quantity. In the polymerization of polyaniline, aniline undergoes a chain reaction, resulting in the formation of macromolecules. In a typical synthesis of polyaniline, 1 mL aniline, 3 mL HCl, and 16 mL milli Q water were mixed in a beaker using a magnetic stirrer and kept undisturbed for 3 h at temperature 1–5 °C and labeled as solution A. In another beaker, the 0.1 M of ammonium persulfate was prepared and labeled as solution B. After 3 h, solution B was added to solution A under constant stirring. At this step, the oxidation of aniline takes place under the action of ammonium persulfate. During this process, cations are generated in an aniline molecule, thus initiating the growth of polyaniline. The finally obtained suspension was continuously stirred for 3 h while maintaining a temperature of 1–5 °C. The polyaniline was synthesized at a lower temperature because a lower temperature favors the synthesis of longer fibers and ordered arrangements, resulting in higher electrical conductivity. The obtained solution was washed with DI water followed by drying at room temperature for 24 h. The schematic for the synthesis of polyaniline is shown in Figure 8.

### 3.3. Characterization

Field emission scanning electron microscopy (FESEM) (JEOL JSM-7600F) from Tokyo, Japan was used to examine the morphology of polyaniline nanofibers. For TEM images, a transmission electron microscope (TEM/HRTEM, JEOL, JEM-2100F) from Tokyo, Japan was used and operated at 120 kV for nanocomposite samples. The phase and crystallinity of synthesized polyaniline nanofibers were examined using the X-ray diffraction technique (XRD) with D8 AαS Advance X-ray diffractometer from Cambridge, London with Cu K radiation (λ = 1.54156 Å) in the range of 5 to 90°. Fourier transform infrared (FTIR) spectroscopy (ATR-FT-IR model “Nicolet IS 10”) from New Jersey, United States with the specular reflectance accessory was used to quantify the surface bonding. The FTIR spectrum of the polyaniline thin film was recorded in the range of 400–2000 cm^−1^. The thin film was prepared by dissolving the polyaniline in DMF and drop-casting the solution on the glass slide of size 1 × 1 cm^2^. Raman spectroscopy was performed on an HR800 UV Raman microscope (Horiba Jobin-Yvon) from Lyon, France. The UV-visible spectroscopy model no. V-670 (JASCO) from Maryland, United States was used to analyze the optical properties of synthesized polyaniline nanofibers. The UV absorbance spectrum of polyaniline was recorded by dissolving the polyaniline into the ethanol and sonicate for 15 min for uniform dispersion. The spectrum was recorded in the range of 300–900 nm with the quartz cuvette of path length 1 cm.

### 3.4. Electrochemical Measurement

The electrochemical detection of glucose was performed using three electrode auto lab workstations potentiostat (VersaSTAT 3, Princeton Research, Princeton, NJ, USA) with Ag/AgCl and platinum wire as reference and counter electrode, respectively. The working electrode of diameter 2 mm and area 3.142 mm^2^ has been modified with the synthesized PANI and was used as a modified working electrode for measurements. The Teflon-coated platinum electrode was gradually washed with sulfuric acid, sodium hydroxide, and acetone to remove the impurities, followed by ultrasonication for 15 min. The synthesized polyaniline nanofiber (100 mg) was dispersed in ethanol (100 mL) under the sonication. The solution was then dropped cast (2 µL) on the platinum electrode, followed by drying at 60 °C for 1 h. The electrochemical measurements were performed in 0.10 M phosphate buffer solution (pH 7). The cycles were repeated with the bare electrode and modified electrode without glucose until they were overlapped to ensure that the response of the electrode was only due to the glucose. The 10 cycles of CV were run before starting the measurements to activate the electrode. After activation, the appropriate potential range for improved sensing was selected, and electrochemical detection of the glucose was performed. The electrochemical impedance spectroscopy was performed with DC potential with the frequency range of 1–10^5^ Hz and amplitude of 1 mV.

## 4. Conclusions

In this study, we present an electrochemical glucose sensor based on an electrode modified with polyaniline nanofibres. Polyaniline was used to create the electrode material, which was then examined using X-ray diffraction, scanning electron microscopy, high-resolution transmission electron microscopy, Fourier transform infrared spectroscopy, and UV-visible spectroscopy. The lower limit of detection for glucose by the created electrochemical sensor was 10.6 µM. Using both cyclic voltammetry and electrochemical impedance spectroscopy, it was discovered that the linear detection range of the electrochemical detection was 10 µM to 1 mM. The potential use of polyaniline as an electrode material for the electrochemical detection of glucose is confirmed by the great stability and sensitivity of the electrode. For seven days, the stability of the same electrode was examined. In the presence of interferences, the electrode exhibits good stability for glucose sensing. PANI nanofiber-based sensors offer the advantage of scalability and cost-effectiveness, making them viable candidates for large-scale production and commercialization. By utilizing scalable fabrication techniques and cost-effective materials, our platform holds promise for widespread adoption and accessibility.

## Figures and Tables

**Table 1:** Summary of electrochemical detection of glucose using polyaniline with detection parameter.

Electrode	Method	LOD	Selectivity	Linear Range	Ref.
**Polyaniline on graphite electrode**	Enzymatic	1 mmoldm^−3^	No	1–5 mmol dm^−3^	[68]
**Polyaniline (PANI) nanofiber dendrites on platinum electrodes**	Enzymatic (amperometric analysis)	100 nM	Yes	50 µM–12 mM	[69]
**Polyaniline-coated graphite**	Non-enzymatic (Amperometric Analysis)	0.01 µM	No	0.01–0.1 µM	[70]
**Polyaniline nanofibers and silver nanoparticles**	Non-enzymatic (chronoamperometry)	1.3 µM	Yes	100 μM to 10 mM	[71]
**Molecularly imprinted polyaniline electrode**	Non-enzymatic	1.0048 mM	No	2.2 to 11.1 mM	[72]
**Polyaniline nanowires**	Enzymatic	0.05 mM	Yes	0–8 mM	[73]
**Polyaniline-activated carbon**	Amperometric analysis	5.0 × 10^−8^ M	Yes	5.0 × 10^−7^–1.0 × 10^−5^ M	[74]
**Polyaniline-gold** **nanoparticles**	Non-enzymatic	0.1 mM	Yes	0.3–10 mM	[75]
**Polyaniline nanofibre on platinum electrode**	CV	10.6 µM	Yes	10 µM–1 mM	**This work**

## Data Availability

The data presented in this study are available from the corresponding authors upon reasonable request.
